# Census of halide-binding sites in protein structures

**DOI:** 10.1093/bioinformatics/btaa079

**Published:** 2020-02-05

**Authors:** Rostislav K Skitchenko, Dmitrii Usoltsev, Mayya Uspenskaya, Andrey V Kajava, Albert Guskov

**Affiliations:** b1 Institute BioEngineering, ITMO University, Saint-Petersburg 197101, Russia; b2 Centre de Recherche en Biologie cellulaire de Montpellier (CRBM), UMR 5237 CNRS, Universite Montpellier, Montpellier 34293, France; b3 Groningen Biomolecular Sciences & Biotechnology Institute, University of Groningen, Groningen 9747 AG, the Netherlands

## Abstract

**Motivation:**

Halides are negatively charged ions of halogens, forming fluorides (F^−^), chlorides (Cl^−^), bromides (Br^−^) and iodides (I^−^). These anions are quite reactive and interact both specifically and non-specifically with proteins. Despite their ubiquitous presence and important roles in protein function, little is known about the preferences of halides binding to proteins. To address this problem, we performed the analysis of halide–protein interactions, based on the entries in the Protein Data Bank.

**Results:**

We have compiled a pipeline for the quick analysis of halide-binding sites in proteins using the available software. Our analysis revealed that all of halides are strongly attracted by the guanidinium moiety of arginine side chains, however, there are also certain preferences among halides for other partners. Furthermore, there is a certain preference for coordination numbers in the binding sites, with a correlation between coordination numbers and amino acid composition. This pipeline can be used as a tool for the analysis of specific halide–protein interactions and assist phasing experiments relying on halides as anomalous scatters.

**Availability and implementation:**

All data described in this article can be reproduced via complied pipeline published at https://github.com/rostkick/Halide_sites/blob/master/README.md.

**Supplementary information:**

Supplementary data are available at *Bioinformatics* online.

## 1 Introduction

Halides are the common anionic forms of halogens, in which the latter interact with the less electronegative atoms, thus forming fluorides (F^−^), chlorides (Cl^−^), bromides (Br^−^) and iodides (I^−^). In this work, we are not considering astatides (At^−^), due to its unstable (radioactive) nature and not known biological functions. In the biological context, halides are often considered merely as the components of buffers to maintain certain ionic strength of a solution, this is especially true for Cl^−^ as it is the most common counterion. Iodides owing to its electron-rich configuration [Kr] 4d^10^5s^2^5p^6^ have found an interesting niche in the macromolecular X-ray crystallography as a phasing agent to solve the phase problem (*vide infra*). Fluoride seems to be a rather exotic anion, for example currently only 67 entries contain fluoride as a ligand in the Protein Data Bank (PDB) (i.e. <0.05% entries), and fluorides are typically rather incorporated into the scaffold of other ligands, i.e. drugs or inhibitors to fine-tune their properties. Bromide seems to have a few highly specialized functions (discussed below) and similarly to iodide can be used for phasing.

For the purpose of this work on characterization of biologically relevant halide-binding sites, we focus only on ionic forms of halides and not on covalent ones. The former are characterized by −1 charge and large radii: 1.19, 1.67, 1.82 and 2.06 Å for F^−^, Cl^−^, Br^−^ and I^−^, respectively (Shannon, 1976).

### 1.1 Fluoride

The biological role of fluoride is typically reviewed in the context of dentistry, as it has been shown as an excellent anti-caries agent (Aoun *et al.*, 2018; Wong *et al.*, 2011). It has a dual role, as it reinforces enamel via slowdown of demineralization and promotion of remineralization via formation of fluorohydroxyapatite (Fincham *et al.*, 1999; Kanduti *et al.*, 2016), but also via suppression of mouth bacteria by inhibiting certain intracellular enzymes, leading to the decreased production of lactic acid and hence lowering the risk of caries formation (Buzalaf *et al.*, 2011). Clearly, taking into account such an inhibitory function, the valid question is the safety of fluoride for humans, especially since in many countries tap water and table salt come fluorinated. According to the current research and guidelines, there is no actual risk for humans under normal circumstances, apart for those who live in the areas, where water is naturally enriched in fluoride—some parts of India, China and Africa continent. The prolonged exposure to the excess of fluoride might lead to the disorders, such as skeletal fluorosis, dental fluorosis and kidney failure (Aoba and Fejerskov, 2002; Perumal *et al.*, 2013; Weatherell, 1969).

However, the environmental levels of F^−^ of 10–100 µM found in soil and water are toxic for many organisms, hence several transport systems to expel fluoride from cells have evolved. One of them is Fluc proteins, which can be found in prokaryotes and lower eukaryotes (Stockbridge *et al.*, 2013). Flucs are anion channels, which are highly specific to F^−^ over Cl^−^ (Stockbridge *et al.*, 2015). The other major class of F^−^ transporting proteins is a subfamily of CLC chloride transporters, strictly confined to bacterial species only, so-called CLCF F^−^/H^+^ antiporters, which couple F^−^ efflux to a proton gradient (Brammer *et al.*, 2014). The selectivity of these transporters for F^−^ over Cl^−^ is also extremely high (Stockbridge *et al.*, 2012).

Clearly the exporters cannot immediately remove the excess of incoming F^−^ flux, hence it readily interacts with intracellular proteins. Among the best characterized targets to which F^−^ binds are F^−^-specific riboswitch, where F^−^ is complexed with Mg^2+^ and RNA phosphate groups (Ren *et al.*, 2012); enolase (Qin *et al.*, 2006), which is an essential enzyme for glycolysis; heme containing proteins and numerous phosphatases (Marquis *et al.*, 2003). Fluorides readily react with aluminum and beryllium, and the formed complexes are highly cytotoxic as they mimic phosphate group hence inhibiting numerous enzymes, which exert ATPase or GTPase activity (Li, 2016). In eukaryotic cells, the deleterious effect of exposure to fluoride is even more dramatic, as fluoride interferes with the cell cycle, respiration, gene expression, oxidative stress and G protein activation (Mendoza-Schulz *et al.*, 2009; Murthy and Makhlouf, 1994; Strunecka *et al.*, 2007).

It is important to note that in many cases the inhibition effect might be caused not via binding of fluorides to proteins, but also as an effect of acidification of cytoplasm, as F^−^ might play a role of transmembrane proton carrier (Barbier *et al.*, 2010; Eisenberg and Marquis, 1981).

The inhibitory effect of fluoride complexes with aluminum and beryllium was quickly recognized as an invaluable tool for structural studies as it allows stabilization of intermediate states. Using such complexes, numerous enzymes as well as several membrane transporters were trapped in specific conformations, e.g. maltose uptake transporter MalFGK2 (Oldham and Chen, 2011) and calcium ATP-ase (Toyoshima *et al.*, 2004).

### 1.2 Chloride

Chloride is a ubiquitous anion both in the environment and in cells, and it plays important roles in all kingdoms of life. Its most common role is as a counterion for sodium and potassium, in combination with which they form an electrolyte mix essential to maintain the concentration and charge differences across cell membranes. The second most important role of chloride (especially in certain bacteria and archaea) is as an osmolyte during osmoadaptation (Saum and Müller, 2008). In a high saline milieu, halophilic and halotolerant microorganisms accumulate up to molar concentrations of chloride (Lanyi, 1974; Oren, 1986). Interestingly some of these organisms (e.g. *Halobacillus halophilus*) are strictly dependent on chloride for their growth, as Cl^−^ is directly involved in regulation of transcription and translation of several essential proteins (Hänelt and Müller, 2013; Roeßler and Müller, 2001; Roeßler *et al.*, 2000). Interestingly, some bacteria which are not normally halotolerant, are capable of growth in the high salt medium, but only if the counterion is a chloride, implying the involvement of chloride in the osmoadaptation also in these species and/or regulation of sodium export (Galinski, 1995). In higher organisms, Cl^−^ also plays important roles. For example, in plants chloride is required for turgor generation and the regulation of cell volume, as well as for generation of Cl^−^ currents (Wege *et al.*, 2017). In photosynthetic organisms (hence also including cyanobacteria and algae), chloride ion plays an essential role in photosystem II function, namely it facilitates the proton flux from the oxygen evolving complex to the thylakoid lumen (Brahmachari *et al.*, 2017; Guskov *et al.*, 2009; Kawakami *et al.*, 2009). In mammals, chloride in the form of hydrochloric acid maintains the very acidic pH of gastric juice (pH 1.5–3.5) necessary to unfold consumed proteins, to activate digestive enzymes and to kill microorganisms susceptible to such acidic environments (Freeman and Kim, 1978; Lu *et al.*, 2010). Furthermore, there are numerous human proteins, where specific binding sites for chloride were revealed and/or which are shown to be affected upon interaction with chloride. Among these α-amylase (Aghajari *et al.*, 2002), angiotensin-converting enzyme I (Yates *et al.*, 2014), hemoglobin (Prange *et al.*, 2001), kinases (Chen *et al.*, 2019), acute myeloid leukemia-1 transcription factor (Wolf-Watz *et al.*, 2001) and many others.

To regulate the flux of Cl^−^, numerous chloride channels and transporters have evolved. The most studied chloride transporting proteins belong to the chloride channel (CLC) family (Jentsch and Pusch, 2018) and chloride intracellular ion channel (CLIC) family (Argenzio and Moolenaar, 2016).

CLC proteins are integral membrane proteins, residing either in plasma or intracellular membranes and encompassing both channels and transporters (Accardi, 2015; Accardi and Picollo, 2010). They are involved in the control of excitability during muscle contraction, acidification of endosomes and lysosomes, and epithelial transport (Jentsch, 2007; Pedersen *et al.*, 2016; Piwon *et al.*, 2000; Smith and Lippiat, 2010). Intriguingly all members form dimers with a separate translocation pathway.

Malfunctions of CLC proteins cause severe diseases, such as myotonia congenita (Imbrici et al., 2015a, b), neuronal ceroid lipofuscinosis (Poët *et al.*, 2006; Yoshikawa *et al.*, 2002), Dent’s disease (Devuyst and Thakker, 2010) and Bartter syndrome (Andrini *et al.*, 2015; Cunha and Heilberg, 2018).

CLIC proteins are quite unique, since they can exist both in soluble and membrane-embedded forms. They are not located in the plasma membrane but abundant in intracellular organelles (Gururaja Rao *et al.*, 2018). They are involved in signaling (Argenzio and Moolenaar, 2016), endosomal trafficking (Argenzio *et al.*, 2014), phagosomal acidification (Jiang *et al.*, 2012), angiogenesis (Ulmasov *et al.*, 2009), actin-dependent membrane remodeling (Berryman and Bretscher, 2000) and other intracellular processes (Littler *et al.*, 2010).

Another well-studied chloride channel is cystic fibrosis transmembrane conductance regulator (Hwang *et al.*, 2018; Liu *et al.*, 2017, 2019). It is an ATP-gated chloride channel evolved from ABC transporter scaffold. Mutations rendering this protein defunct lead to the increased viscosity of mucus on membranes (e.g. in the lungs) which can be lethal. There are several more families of chloride channels, such as calcium-activated chloride channels (Berg *et al.*, 2012), maxi Cl^−^ channels (Sabirov and Okada, 2009) and volume-regulated chloride channels (Osei-Owusu *et al.*, 2018), which are beyond the scope of this work.

### 1.3 Bromide

There is no solid evidence for a certain role of bromide in prokaryotes, although there is a large class of marine and soil microorganisms capable of oxidizing methyl bromides via transmethylation or monooxygenase pathway (Hoeft *et al.*, 2000). For the majority of microorganisms though, bromide is toxic at high concentrations, and in fact it is used as a disinfectant agent, typically in the form of hypobromous acid.

In eukaryotes, the role of bromide was for a long time rather elusive and only recently it has been established that it is essential for the assembly of collagen IV scaffolds during tissue development (McCall *et al.*, 2014). Furthermore, bromide is a preferred substrate for eosinophil peroxidases (Henderson *et al.*, 2001; Thomas *et al.*, 1995), which catalyze the conversion of bromide to hypobromous acid for the host defense.

Bromide is localized mainly extracellularly, and its concentration seems to be tightly regulated (Barratt and Walser, 1969). Bromide deficiency leads to diminished tissue growth and causes failures in tissue development and remodeling (McCall *et al.*, 2014). However, at excess, bromide can cause bromism—the collective name of several neurological disorders caused by the neurotoxic effect of prolonged consumption of bromide (Frances *et al.*, 2003; Lugassy and Nelson, 2009; van Leeuwen and Sangster, 1987).

Interestingly, some marine algae accumulate large amounts of bromide (and iodide) but in the cell wall and usually not in the cytosol (Küpper *et al.*, 2018; Saenko *et al.*, 1978). Their genomes encode a specific set of proteins to deal with halides, such as haloacid and haloalkane dehalogenases as well as vanadium haloperoxidase (Almeida *et al.*, 2001; Kunka *et al.*, 2018). The latter enzyme is responsible for production of methyl halides. The accumulation of bromide is probably a consequence of the relative abundance of this anion in seawater: the average concentration of Br^−^ is ∼65 mg l^−1^, whereas F^−^ and I^−^ are ∼1 mg l^−1^ (however, Cl^−^ is predominant with concentration of 19 000–23 000 mg l^−1^). Surprisingly despite the concentration of Br^−^ is 300 times lower than that of Cl^−^, in many cases it is rather bromide than chloride (or their combination) which is used for organohalogen production (Cabrita *et al.*, 2010).

In the world of structural biology, bromide has caught an eye due to its phasing potential—with 36 electrons and easily accessible X-ray absorption edge (K edge ∼0.92 Å), it is a good choice for single or multi-wavelength anomalous diffraction (SAD/MAD) phasing (Dauter and Dauter, 2007). In the easiest application, a crystal should be shortly soaked in a cryoprotectant containing bromide just before the flash freezing in liquid nitrogen (Dauter *et al.*, 2000). Bromide ions will quickly diffuse via the solvent channels and settle within the ordered solvent shell around the protein surface (Dauter and Dauter, 1999, 2001).

### 1.4 Iodide

Iodide is one of the largest monoatomic anions and one of the heaviest elements utilized by living organisms. In vertebrates, it is utilized for the production of growth-regulating thyroid hormones (thyroxine and triiodothyronine), which are essential regulators of virtually nearly all processes during different life phases (Bernal *et al.*, 2015; Eales, 1997; Senese *et al.*, 2014).

Uptake of iodide into thyroid occurs via the sodium iodide transporter (SLC5A5) (Darrouzet *et al.*, 2014) residing in the basolateral membrane of thyroid follicular cells. The transport is active and the inward translocation of sodium down its electrochemical gradient is coupled to inward translocation of iodine against its electrochemical gradient.

Other organisms such as algae, zooplankton and plants are capable to accumulate iodine/iodide as thyroid hormone precursors, which can be used as developmental regulators. Many bacteria are capable of extracting necessary iodine/iodide from the host environment (DiStefano *et al.*, 1993). Marine microorganisms are especially agile in iodide accumulation as they are capable to reduce inorganic iodate (the most thermodynamically stable form) to iodide and produce numerous iodinated organic compounds (Küpper *et al.*, 2018). However, many other bacteria, both aerobic and anaerobic, are also able to convert iodate to iodide. It seems that even in the absence of highly specialized enzymes of thyroid gland, iodothyrosines can form spontaneously and due to its reactivity play a crucial role in cell–cell signaling (Cahnmann and Funakoshi, 1970; Crockford, 2009).

Therefore, a plausible scenario is that during evolution iodine/iodide reacting with tyrosines might had been recruited as a potent signaling molecule somewhat after last universal common ancestor (Crockford, 2009).

Some macroalgae (kelp) developed an extreme concentrating capacity for iodine, e.g. *Laminaria digitata* can concentrate up to 30 000 more of iodide in its apoplasts compared to iodide concentration in seawater (Küpper *et al.*, 2011). Such accumulation leads to a buildup of antioxidant reservoir that is mobilized during oxidative stress. Iodide can scavenge not only H_2_O_2_ and ozone, but also hydroxyl radicals and superoxides (Küpper *et al.*, 2008, 2018).

Iodine deficiency in humans is well documented and leads to the numerous mental and physical developmental delays (Zimmermann, 2011) and currently up to two billion people are affected worldwide according to World Health Organization. The iodized table salt turned out to be an excellent tool to compensate for insufficient uptake of iodine in affected populations. The large excess of iodine, however, can be toxic, especially in the case of selenium deficiency, when the function of Se-containing antioxidative enzymes is impaired.

One can exploit anomalous signal of iodides bound to proteins the same way as it is done with bromide.

The X-ray absorption edge for iodide is not readily accessible (L–I edge ∼2.39 Å), however, even far from it (at wavelengths of ∼1.8 Å, which are accessible at modern synchrotrons), the anomalous signal is roughly three times higher than for bromide. The fast iodide soaking before cryo-freezing turned out to be successful phasing technique for numerous soluble proteins (Abendroth *et al.*, 2011). Recently it has been proposed that iodide SAD phasing might be universally applied to membrane proteins (Melnikov *et al.*, 2017) as their positively charged residues found at the hydrophobic–hydrophilic interface need a compensatory negative charge, hence increasing the odds of (ordered) binding of iodides at these areas.

Additionally, all four halides were shown to restore the oxidative redding (green to red photoconversion) in fluorescent proteins (Bogdanov *et al.*, 2016), adding another possible application role in the fine tuning of properties in this important class of proteins.

Taking into account the importance of halides in the biochemistry of all life forms and possible applications we became intrigued whether there are certain patterns of halide binding to proteins—preferred amino acids involved in the binding sites and its geometry. We have analyzed all the PDB entries, containing halides, available on August 23, 2019, in the PDB and revealed the following patterns.

## 2 Materials and methods

### 2.1 Data acquisition and filtering

X-ray data analysis of protein structures containing fluoride, chloride, bromide and iodide, which are coordinated only by protein without ligands, were obtained from the PDB using Biopython (module Bio.PDB). List of entries was obtained with an advanced search (Search parameters: Chemical name—chloride/bromide/iodide/fluoride, Name—Equals, Polymeric type—Any). 67, 12 686, 455 and 864 entries were obtained for F^−^, Cl^−^, Br^−^ and I^−^, respectively. PDB structures obtained by NMR, powder diffraction, cryo-electron microscopy and neutron diffraction were excluded. For entries with the same name of proteins, those with the highest resolution were selected. Entries with a resolution of lower than 2 Å were excluded. The final non-redundant dataset includes 25, 3229, 206 and 246 structures with F^−^, Cl^−^, Br^−^ and I^−^, respectively. Analysis was performed with and without water molecules. If a PDB entry contained several identical halide sites (i.e. the case of homologous sites), only one site was taken and the rest were excluded from further study (the similarity threshold was arbitrary set at 0.5 Å rmsd). Sites containing non-protein atoms such as small ligands from HETATM or DNA/RNA from ATOM field were excluded. Sites consisting of several chains (∼10% of all entries) have not been taken into account.

### 2.2 Calculations of distances, angles and accessible surface area

Distances and angles were calculated for each atom within a sphere with a radius of 5 Å around the halide using NumPy. Interactions of halides with carbon and hydrogen atoms were not considered. Interactions were examined both in the absence and in the presence of water molecules. In cases when the halide had more than one coordinating atom, the angles were calculated between two vectors: halide—the nearest atom and halide—atom. Fractional accessible surface area (fASA) of each halide atom was calculated with FreeSASA (Mitternacht, 2016) as a ratio between ASA of the halide within the protein structure and ASA of the sphere with the radius including halide radius (1.19, 1.67, 1.82 and 2.06 Å for F^−^, Cl^−^, Br^−^ and I^−^, respectively) and water molecule radius (1.4 Å). fASA allows to distinguish buried versus surface-bound halides.

### 2.3 Workflow reproducibility

Since the PDB is continuously expanding, the addition of new entries will make our analysis more robust. Snakemake pipeline was constructed by using Python programming language to provide the ability to reproduce the results of this study. The pipeline covers all stages of the current study from downloading of halide-bound protein structures from the PDB to generation of the output files and graphical output in the course of several minutes. The output is tab-separated file with information about each halide atom in PDB structures. Additionally, there is an anaconda-environment file, which provides instructions for the installation of the dependencies required for the workflow. The pipeline is available at https://github.com/rostkick/Halide_sites/blob/master/README.md.

## 3 Results

The distribution of entries in PDB containing different halides is not equal, there are only 67 entries with fluoride and over 12 000 for chloride; for bromide and iodide, there are 455 and 864 entries. After applying strict selection criteria (see Section 2), the resulting working dataset contains 25, 3229, 206 and 246 entries for F^−^, Cl^−^, Br^−^ and I^−^, respectively.

### 3.1 Bimodal distributions of distances between halides and atoms of amino acid residues

**Fig. 1.**
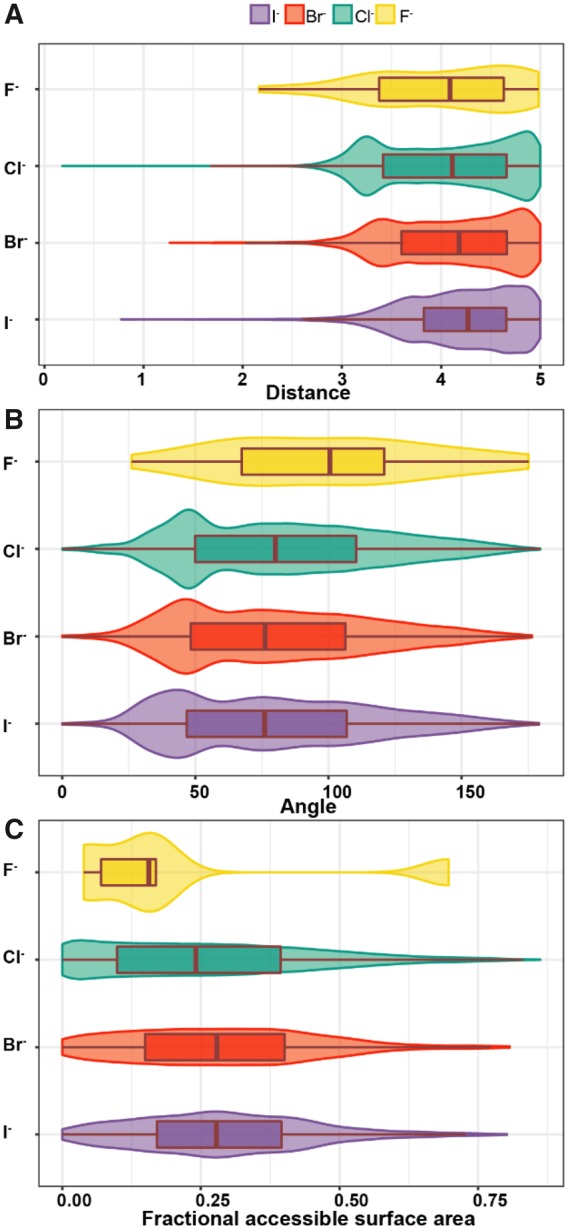
Halide-binding sites in proteins. Distribution of (**A**) distances between halide and coordinating atoms, (**B**) angles between two vectors (halide-nearest-coordinating atom, halide-coordinating atom) and (**C**) fractional ASA values

For all four halides, the median distance between an anion and docking residue is about 4.16 Å (Fig. 1A), with the smallest value of 4.09 Å for fluoride followed by 4.11 Å for chloride, 4.17 Å for bromide and the largest value of 4.28 Å for iodide reflecting difference in their ionic radii (*vide supra*). Such distances are indicative that the bound anions are either partially or completely dehydrated, as their hydrated radii are within 3.3–3.5 Å (Ayala *et al.*, 2003; Paula *et al.*, 1998).

However, if we look at the distribution of distances (Fig. 1A), the largest deviation is observed for chloride and iodide, with the distances going closer than 1.5 Å—these must be clear outliers as at such a short distance the repulsion of atoms is inevitable. The possible explanation of such a spread is that with the higher number of entries the odds of getting erroneous assignment is also higher; additionally, in the case of chloride since its anomalous signal is very low, it is hard to verify its assignment and it can be easily confused with water molecules. Fortunately, the number of such cases is low.

Surprisingly there is a large number of all four halides with the distances around 5 Å, those represent situations where the anions interact with atoms of residues via a water molecule. With that notion, we decided also to include water molecules in the analysis of interactions. It turns out that iodide tends to be less solvated (Supplementary Fig. S1A), with the preference for two water molecules in its network, whereas Br^−^ and Cl^−^ prefer three water molecules. Furthermore, the distribution of distances and angles in the presence of water molecules has not changed considerably, apart from fluoride (Supplementary Fig. S1B and C). These observations agree well with the Hoffmeister series on Δ*G*_hydration_ energies, where F^−^ is the most and I^−^ is the least strongly solvated anion (Fox *et al.*, 2015).

### 3.2 Angular distribution of coordinating atoms

The distribution of angles between the coordinating bonds is quite wide (Fig. 1B and Supplementary Fig. S1C), reflecting the possible positional errors (which can be of various origins and influenced by data quality and resolution). Nevertheless, Cl^−^, Br^−^ and I^−^ have a noticeable maximum at 45 degrees and also less visible blurred maximum at around 90 degrees, indicative of bipyramidal or octahedral arrangement of binding sites. However, in the presence of water, the maximum is rather shifted toward larger angular values.

### 3.3 Typical compositions of halide-binding sites

The analysis of accessible surface area of bound halides revealed that in the majority of cases, the binding sites form pockets that surround the halides. At the same time, on average, 35% of the halide surface is accessible to the solvent. It means that the binding sites are located on the surface of proteins, apart from fluoride, which seem to be buried deeper inside (Fig. 1C).

**Fig. 2.**
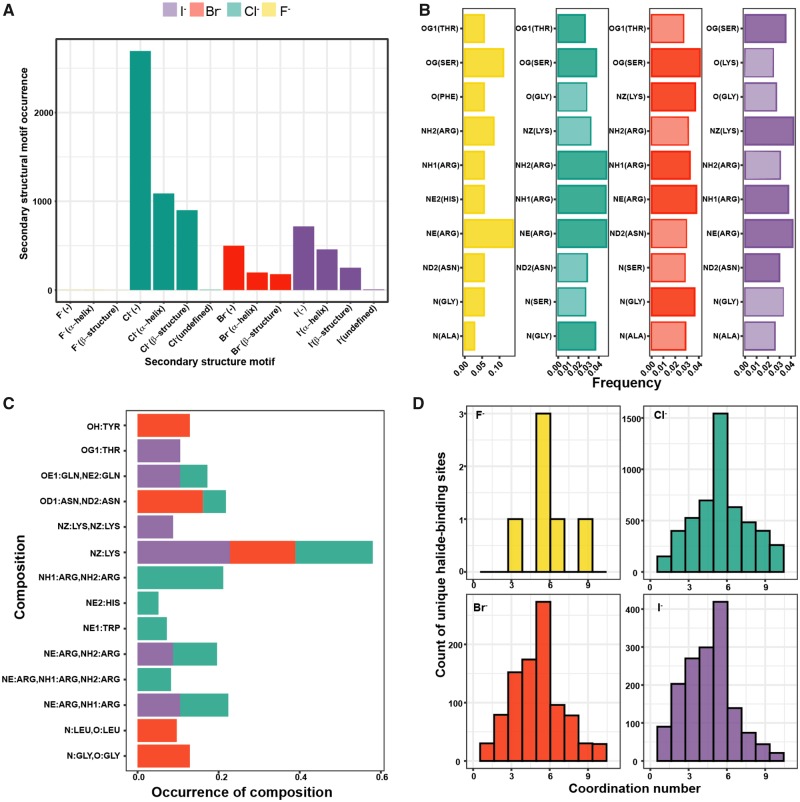
Composition of halide-binding sites in proteins. (**A**) Preference to bind to certain secondary structure elements, namely α-helix, β-strand and – none. (**B**) The amino acid composition of binding sites, (**C**) certain combinations of amino acids in binding sites and (**D**) coordination numbers of halides

We have also analyzed preferences of halides binding to different secondary structure elements (Fig. 2A) and it shows that the binding sites are predominantly located in the loop regions between α-helices and β-strands. At the same time, the α-helices are more frequently found to provide the interacting residues than β-strands.

In terms of amino acid residues which compose the binding sites, there is a strong preference for positively charged amino acid residues for all four halides (Fig. 2B). Arg side chains are the most universal anchor for halide binding via its guanidinium moiety. Interestingly for halide-containing small molecules, this is in general also true (Supplementary Fig. S2), however, in this case, polar Ser, Thr, Asn, Gln and also negatively charged Asp and Glu (especially in case of fluorine- and bromine-containing compounds) also play a significant role. This might be dictated by the exact composition of a small molecule with an incorporated halogen atom.

Polar Ser and Thr (with hydroxyl side chain) and Asn (with carboxamide group) also participate frequently in the coordination of halides (Fig. 2B). However, to our big surprise, there are numerous cases, where negatively or partially negatively charged atoms from Asp and Glu side chains as well as mainchain carbonyl are involved (∼30% of cases). At the physiological pH values, these atoms, in principle, should have repulsion with halides. However, it seems that at least in some structures these interactions are mediated by water molecules. For Br^−^, I^−^ and Cl^−^ sites there is an additional positive charge provided by side chains of Lys via its ε-amino group and in case of F^−^, it is provided by imidazole group of histidine. Interestingly for all halides, the main chain (namely its NH group) of small residues (Ala, Ser and especially Gly), is frequently involved in the interactions (Fig. 2B).

When we checked for the common combinations in the binding sites for various halides, we revealed that some combinations are specific to a certain halide (Fig. 2C). For example, the combination of two Lys side chains occurs only for I^−^, whereas single Lys residue might be a part of Cl^−^, Br^−^ or I^−^ binding sites. The Tyr side chain is unique to Br^−^ binding sites, as well as the combination of two Gly residues, whereas Trp and His side chains are found in Cl^−^ binding sites only.

### 3.4 Coordination numbers of halides in binding sites

The coordination numbers for halides vary a lot (Fig. 2D)—from two, indicating the simplest linear configuration, up to nine, corresponding to either tricapped trigonal prismatic or capped square antiprismatic configuration (Supplementary Fig. S3). However, it turns out that the most common coordination number for all halides is 5 (Fig. 2D), corresponding to trigonal bipyramidal or square pyramidal geometry of the binding sites.

Interestingly, there is a certain correlation between the coordination numbers of halides and the amino acid residues involved in the respective binding sites (Supplementary Fig. S4). For example, Lys residues are way more common in the binding sites of halides with the low coordination numbers, whereas Cys residues are exclusively found in the sites of halides with the high coordination numbers.

## 4 Discussion

Halides are ubiquitous in the environment and impact all living organisms on our planet. Whereas some of them, such as chloride became a universal counterion for positively charged potassium and sodium, and contribute to buildup of electrochemical gradients, others, such as bromide and iodide play rather very defined roles, for example as strong antioxidants. Fluoride is mostly considered as a toxic compound, and many organisms developed fluoride expelling channels, and the only well-documented case where it is actively used as an essential compound is the class of adenosyl-fluoride synthases, catalyzing the formation of a carbon–fluorine bond to S-adenosyl-l-methionine, with the concomitant release of l-methionine (O’Hagan *et al.*, 2002). Furthermore, there are many other enzymes which catalyze the formation of numerous halogenated products, some of which are actively being investigated for their potent pharmacological properties (Jesus *et al.*, 2019; Kasanah and Triyanto, 2019; Rocha *et al.*, 2018).

Considering all the importance of halides in biology, it is surprising that rather little is known about their binding to proteins, apart from the logical suggestion that negatively charged anions will be recruited to the positively charged side chains via the formation of ion pair as dictated by the so-called ‘law of matching water affinities’ (Collins, 2006). Although our extensive analysis confirms this general observation, it additionally reveals certain patterns and preferences among four halides for its binding partners. For example, the occurrence of Arg side chain might be a general flag for the halide-binding site, however, the presence of other positively charged residues such as Lys and His, or polar residues can hint to the certain halide.

Based on amino acid composition of the binding sites, interaction lengths and angles, fASA and coordination numbers, it should be feasible to distinguish halides from other ions, however, the predictor is certainly not powerful enough to unambiguously assign the exact identity of a halide in question (Supplementary Fig. S5). In cases when the anomalous data are present, such an identification can be done more properly.

Furthermore, our analysis is of value only for the proteins with the structural models obtained; it is not possible to highlight the putative halide-binding sites based on the amino acid sequence only. At the same time since in many cases the formation of binding sites involves conformational changes of interacting side chains, the apo structures are not very likely to yield proper predictions. Nevertheless, the revealed patterns might be used either for fast evaluation of available PDB models in general to verify the assignments of ions in the absence of any other source of information and/or engineering of halide-binding sites in proteins.

The assembled pipeline can also be used just for analysis purposes. Our present analysis of the dataset of halide-binding sites obtained by using this pipeline demonstrates its usefulness and opens an avenue for more detailed future studies on halides binding to proteins.

## Funding

This work was supported by the Government of the Russian Federation through the ITMO Fellowship and Professorship Program and by the grant 08-08.

*Conflict of Interest*: none declared.

## Supplementary Material

btaa079_Supplementary_Data
