# Supplementary material for: Census of halide-binding sites in protein structures
Source: Bioinformatics. 2020 Feb 5;36(10):3064–71. doi: 10.1093/bioinformatics/btaa079 (PMC7214031; doi:10.1093/bioinformatics/btaa079)
Supplement: btaa079_Supplementary_Data [file btaa079_supplementary_data.docx]

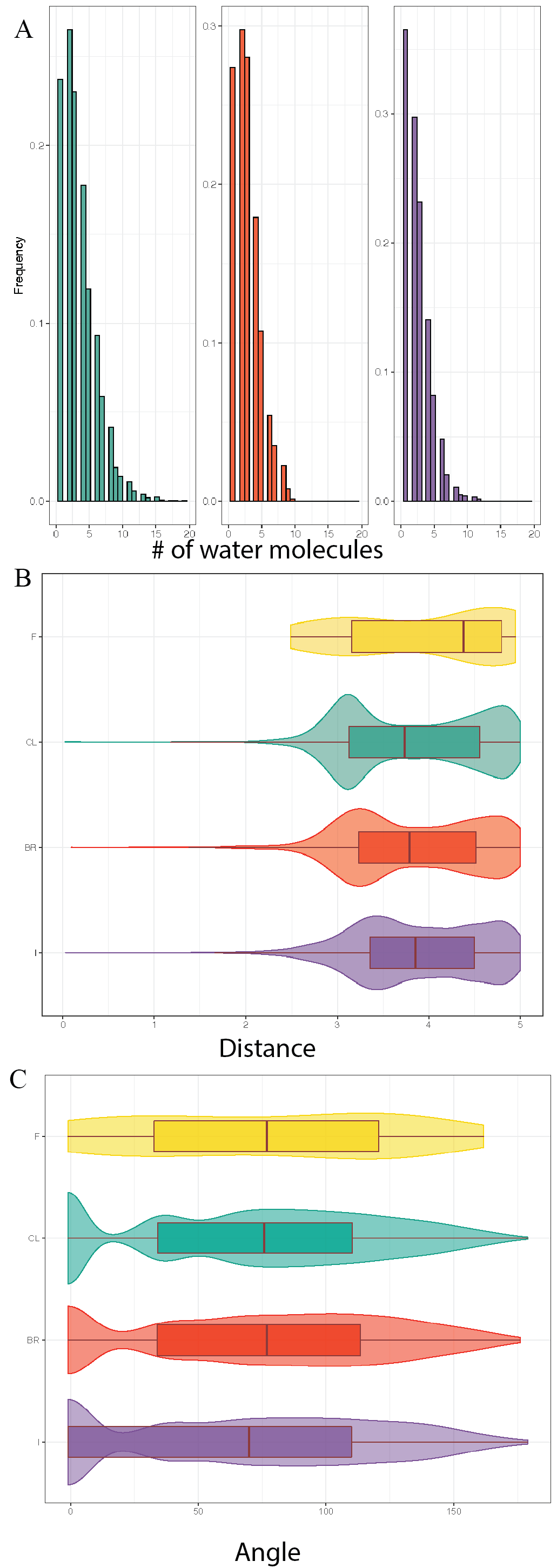


Supplementary Figure 1. Halide binding sites in proteins (analysis in the presence of water). (A) number of observed bound water molecules, (B) distribution of distances and angles between halide and coordinating atoms, (C) angles between two vectors (halide-nearest

coordinating atom, halide-coordinating atom).

Supplementary Figure 2. Comparison of frequency of certain amino acids as binding partners for halides (‘halide per se’, red bars) and halogen-containing (‘contains halide’, black bars) small molecules.

Supplementary Figure 3. Examples of different arrangements in binding sites. Protein is shown in sticks, halides is a sphere. Distances are in Å. (A) trigonal bipyramidal, coordination number 5 (PDB id 2WSL) (B) trigonal prismatic, coordination number 6 (PDB id 1HZJ) (C) capped trigonal prismatic, coordination number 7 (PDB id 1MGY) (D) bicapped trigonal prismatic, coordination number 8 (PDB id 2D8W).


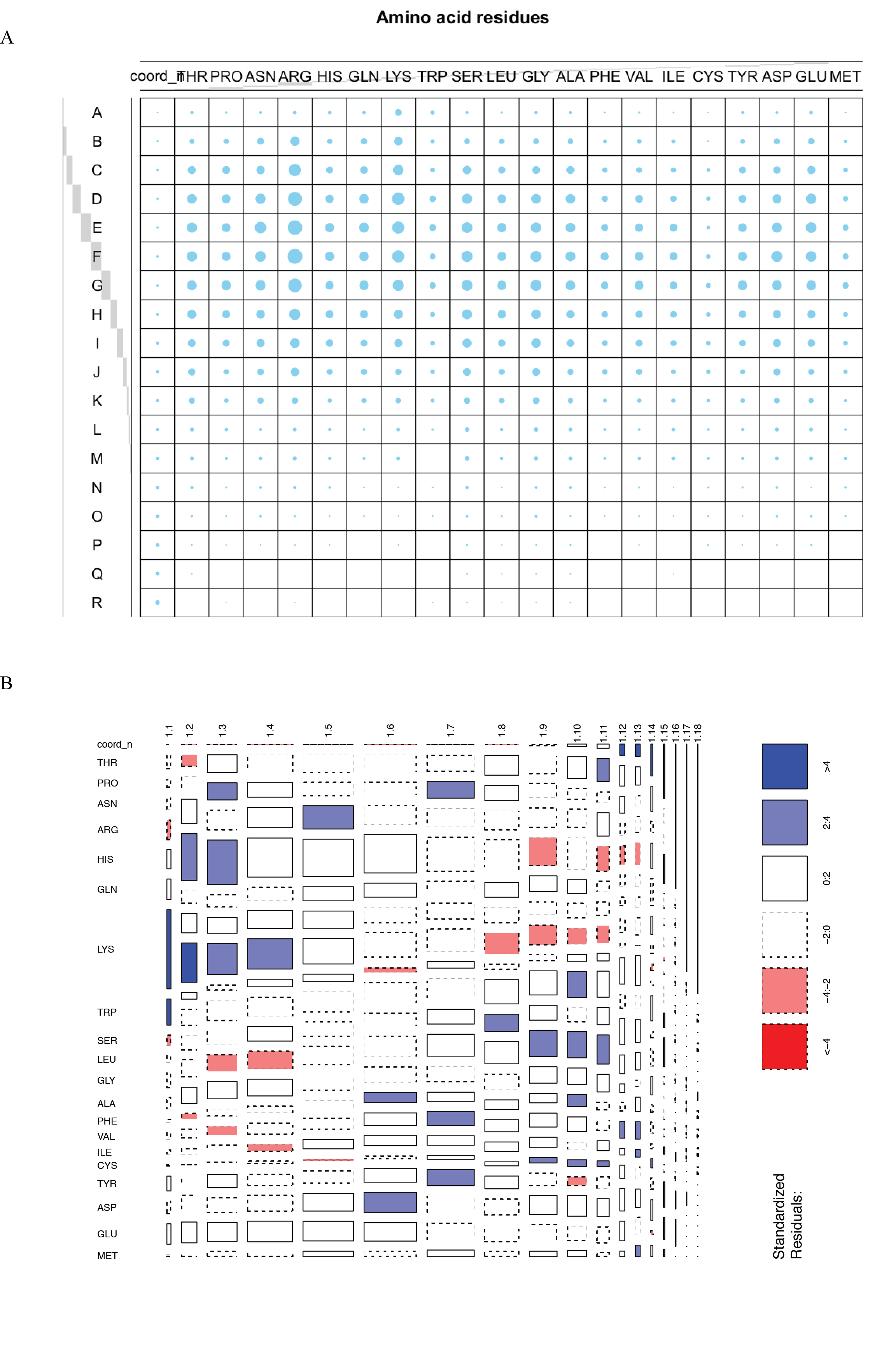


Supplementary Figure 4. Correlation between amino acid residues in the binding sites and halides’ coordination numbers based on Pearson's Chi-squared analysis


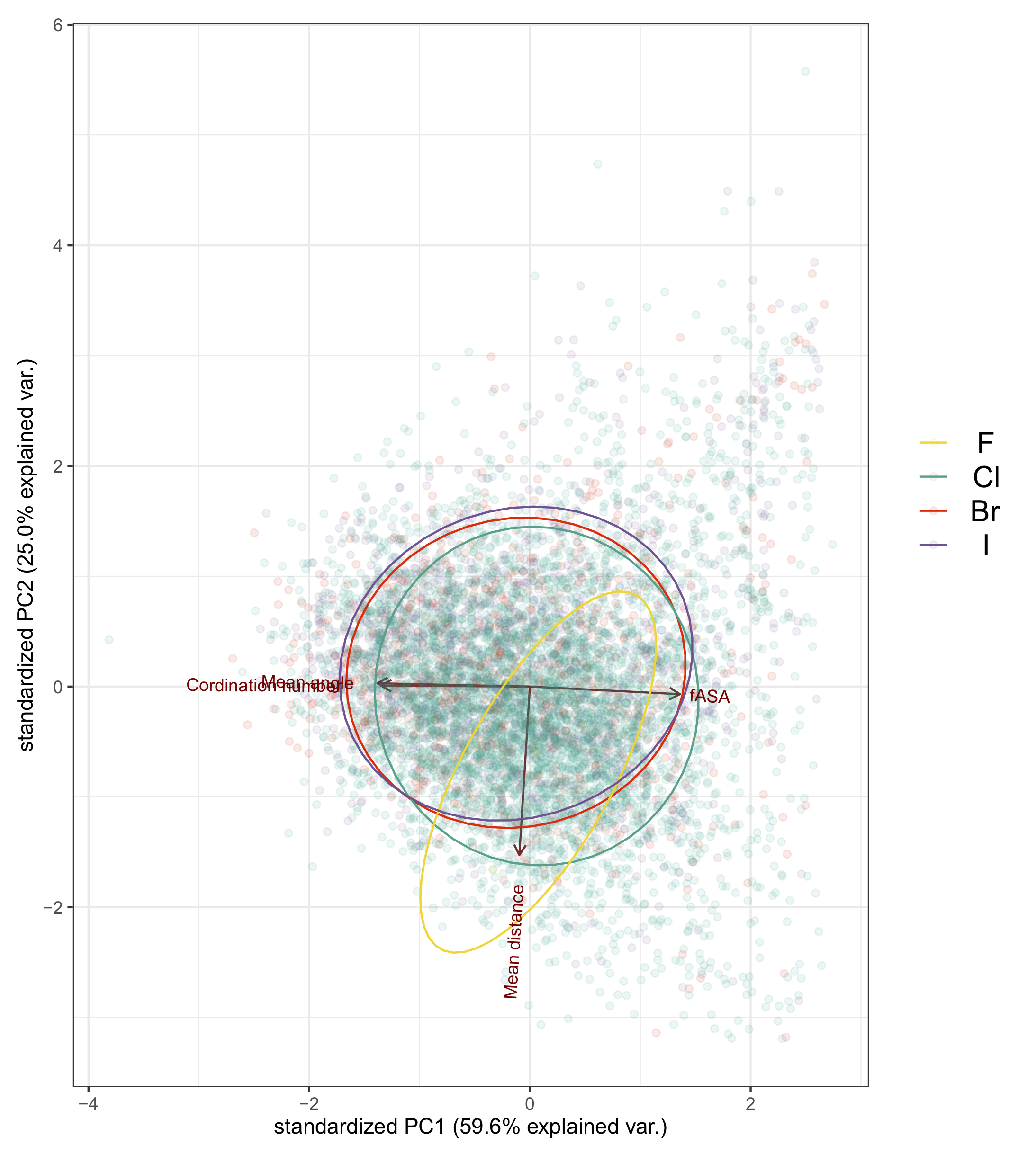


Supplementary Figure 5. Principal component analysis of filtered dataset
